# Surgery of Kerion, a Nightmare for Nondermatologists

**DOI:** 10.1155/2020/8825912

**Published:** 2020-09-15

**Authors:** Vikash Paudel

**Affiliations:** Department of Dermatology, National Medical College, Birgunj, Parsa, Nepal

## Abstract

Kerion is an inflammatory type of tinea capitis characterized by swelling and alopecia of the scalp, which could be mistaken as bacterial infection. It occurs most frequently in children. We report a 10-year-old child whose kerion was misdiagnosed as bacterial abscess and unnecessarily incised. Later, her condition was rediagnosed as kerion based on clinical appearance and potassium hydroxide wet mount. The lesions resolved completely with systemic antifungal treatment, griseofulvin, leaving residual scarring alopecia. The delay in the proper diagnosis and inappropriate treatment of this patient resulted in permanent scarring alopecia. Thus, clinicians must have a high index of suspicion for tinea capitis when dealing with inflammatory scalp lesions.

## 1. Introduction

Kerion is an inflammatory fungal infection of the hair follicle of the scalp, characterized by boggy swelling, purulent discharge, alopecia, and lymphadenopathy. It is more common among children. Different pathogens have been implicated to cause kerion, and the predominant are dermatophytes of genus Trichophyton and Microsporum [1]. Because of its clinical presentation, it might be mistaken for pyoderma, especially an abscess [2, 3].

We report a case of kerion of a child which was misdiagnosed and mismanaged as pyoderma with incision and drainage under local anesthesia, resulting in delayed specific treatment, unnecessary economic burden, risk of anesthesia and surgery, and severe morbidity of scarring alopecia. Thus, we recommend that patients especially children who present with boggy swelling on the scalp should be seen by dermatologists for its appropriate management.

## 2. Case Presentation

It is a story of a 10-year-old female child who presented with mild, tender, itchy, and boggy swelling of the scalp for one-month duration. The lesions started as a small tender nodule over the vertex which gradually increased in size to cover a larger area of vertex. It was associated with areas of hair loss and easily pluckable hairs and infrequent purulent discharge. For this, she visited a nearby surgeon who mistook it as a bacterial abscess and underwent incision and draining under local anesthesia. During surgical procedures, the scalp tissue was very friable to leave a chunk of depression, which could not be sutured and was referred to us for further management.

**Figure 1:**
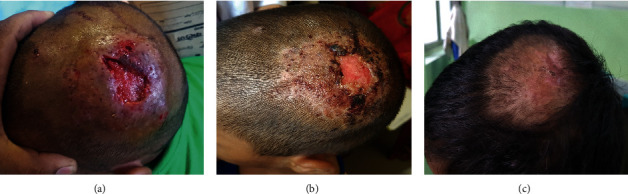
(a) Initial lesion of tinea capitis after surgical incision. (b) Two weeks after starting oral griseofulvin. (c) Eight weeks after starting oral griseofulvin.

**Figure 2:**
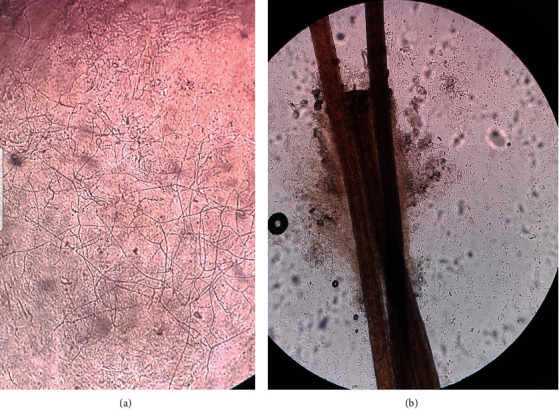
(a)Branching hyphae under KOH wet mount (10x). (b) Ectothrix shown in hair follicle in KOH wet mount (40x).

Examination revealed a localized area of hair loss at the vertex of scalp (6 × 5 cm) with dispersed multiple boggy nodules and polygonal incised ulcer (3 cm × 1.5 cm) with friable edges at the middle of alopecia. Areas of purulent discharge and patchy hair loss were also noted at the periphery of lesions (Figure 1(a)). The left anterior cervical lymph nodes were palpable and tender. Wood's lamp examination revealed yellow-green fluorescence over the affected area. Potassium hydroxide (KOH) wet mount was performed in hair samples and scalp scrapings, which showed multiple branching hyphae from skin lesion and ectothrix in hair follicles (Figures 2(a) and 2(b)). A provisional diagnosis of kerion with secondary ulcer was made, and the patient was prescribed oral griseofulvin 250 mg daily and ketoconazole shampoo for 6 weeks along with oral flucloxacillin, analgesics, and Condy's crystal solution of 1 : 10000 of potassium permanganate for 1 week. She was advised to follow up frequently until the lesion subsides. Eight weeks later, the lesions healed leaving residual scarring alopecia in the incised region (Figures 1(b) and 1(c)).

## 3. Discussion

Tinea capitis is a common and easily transmitted childhood fungal scalp infection. Kerion is a type of inflammatory tinea capitis associated with tender boggy swelling, purulent discharge, alopecia, and regional lymphadenopathy [1]. It is common among children but can be seen from newborn to elderly [4]. Its improper management could lead to permanent scarring alopecia. The most common pathogen responsible for kerion in humans is *Microsporum* and *Trichophyton*, which are zoophilic dermatophytes. Potassium hydroxide (KOH) wet mount, Wood lamp examination, and dermoscopy aid in diagnosis, whereas the fungal culture helps to identify species which might not be always positive [5]. Because of bogginess of the kerion, discharge, and regional lymphadenopathy, it could be confused with bacterial pyoderma, especially by nondermatologist where the clinical exposure of fungal infection (tinea capitis) is limited. In our patient, the diagnosis of kerion was missed by the surgeon, and thus incision and drainage were unnecessarily performed.

Our diagnosis was based on typical clinical features and KOH mount. Fungal culture and species identification were not possible because of unavailability of advanced laboratories facilities. The standard treatment of tinea capitis is systemic antifungal therapy for two to six weeks with oral terbinafine, griseofulvin, or itraconazole, whereas with griseofulvin, mycological cure and efficacy rates are generally high, so it is commonly preferred [6]. The duration of therapy could be extended until clinical clearance is achieved. Topical antifungal as monotherapy is not recommended as these agents are unable to penetrate the hair follicle adequately. Adjunctive topical therapies such as selenium sulphide or clotrimazole or ketoconazole shampoo or creams reduce disease transmission and shorten treatment duration [7]. Antihistamines might be helpful for pruritus and in reducing dissemination of spores due to scratching. Antibiotic treatment is not recommended unless there is a chance of secondary bacterial infection or lesion is traumatized. There is no role of surgical incision and debridement in the case of kerion [8, 9]. There is a controversial role of oral steroids in management of kerion, and thus they are usually not recommended as part of routine care for kerion [10]. As there is no strong recommendation in the use of oral steroid and the patient had history of surgical manipulation of the lesions, oral steroid was not considered in our case.

As illustrated in this case, kerion could easily be misdiagnosed as a bacterial infection due to its clinical presentation. Only the systemic antifungal therapy is the foundation of treatment, and surgical incision is unnecessary and is associated with hazards. In the absence of laboratory facilities, a trial of oral antifungal agents is justified while monitoring the treatment response. Clinicians should have a high index of suspicion for tinea capitis when dealing with inflammatory scalp lesions.

## Data Availability

No data were used to support this study.
